# Exploring resveratrol dimers as virulence blocking agents – Attenuation of type III secretion in *Yersinia pseudotuberculosis* and *Pseudomonas aeruginosa*

**DOI:** 10.1038/s41598-020-58872-0

**Published:** 2020-02-07

**Authors:** Charlotta Sundin, Caroline E. Zetterström, Duc Duy Vo, Robert Brkljača, Sylvia Urban, Mikael Elofsson

**Affiliations:** 1 Department of Chemistry, Umeå University, SE 901 87 Umeå, Sweden; 2 School of Science (Applied Chemistry and Environmental Science), RMIT University, GPO Box 2476 Melbourne, Victoria, 3001 Australia

**Keywords:** Chemical biology, Drug discovery, Microbiology

## Abstract

Bacterial infections continue to threaten humankind and the rapid spread of antibiotic resistant bacteria is alarming. Current antibiotics target essential bacterial processes and thereby apply a strong selective pressure on pathogenic and non-pathogenic bacteria alike. One alternative strategy is to block bacterial virulence systems that are essential for the ability to cause disease but not for general bacterial viability. We have previously show that the plant natural product (-)-hopeaphenol blocks the type III secretion system (T3SS) in the Gram-negative pathogens *Yersinia pseudotuberculosis* and *Pseudomonas aeruginosa*. (-)-Hopeaphenol is a resveratrol tetramer and in the present study we explore various resveratrol dimers, including partial structures of (-)-hopeaphenol, as T3SS inhibitors. To allow rapid and efficient assessment of T3SS inhibition in *P. aeruginosa*, we developed a new screening method by using a green fluorescent protein reporter under the control of the ExoS promoter. Using a panel of assays we showed that compounds with a benzofuran core structure i.e. viniferifuran, dehydroampelopsin B, anigopreissin A, dehydro-δ-viniferin and resveratrol-piceatannol hybrid displayed significant to moderate activities towards the T3SS in *Y. pseudotuberculosis* and *P. aeruginosa*.

## Introduction

The discovery and introduction of antibiotics is recognized as one of the greatest advances in therapeutic medicine during the 20^th^ century. The possibility to employ antibiotics to directly target essential processes enabled us to take the lead in the arms race with pathogenic bacteria that have plagued humans for centuries. However, we are on the verge of losing this advantage to the ever-evolving microbes that develop and spread antibiotic resistances with alarming speed. To be able to turn the tide, decisive action needs to be taken. It is important to reduce the use of broad-spectrum antibiotics and replace them with pathogen-selective compounds that, in contrast to broad acting agents, do not facilitate acquisition, maintenance and spread of resistance determinants by pathogens and non-virulent bystanders alike. One strategy is to develop therapeutic agents that act on major virulence systems in pathogenic bacteria and thus disarm them and assist the immune defense in clearing the pathogen. The advantage with this strategy is that only the pathogenic bacteria are affected and that the commercial flora is kept intact. In addition, virulence blocking agents are expected to act also on bacterial strains resistant to conventional antibiotics in current use.

One of the important virulence systems in Gram-negative bacteria is the type III secretion system (T3SS). The T3SS is a syringe-like apparatus composed of a basal body spanning the inner and outer membrane of the bacterium and a needle complex protruding from the bacterial surface. Upon host cell contact the system translocates toxins into the cytosol of the eukaryotic cell to induce cell injury, subvert the host defense and promote bacterial proliferation. Antibodies binding to proteins at the tip of the needle structure block T3SS function and promote passive protection in animal infection models^1^. In addition, mutation of the T3SS reduces infection of eukaryotic cells and mice^2,3^. The T3SS is conserved among a large number of Gram-negative pathogens and research over the last decades has demonstrated that the T3SS constitutes a validated target for the development of novel pathogen selective therapeutic agents^4^.

*Yersinia* spp. and *Pseudomonas aeruginosa* are two Gram-negative bacteria that harbor very homologous T3SS, but each causes very different diseases. *Yesinia* constitutes eleven species, of which *Y. entercolitica, Y. pestis*, and *Y. pseudotuberculosis* are pathogenic to humans^5^. *Y. pestis*, the causative agent of bubonic plague, is probably the most well-known of these since it was the pathogen responsible for the Black Death in the middle of the 14th century. *Y. entercolitica* and *Y. pseudotuberculosis* cause inflammation in the gastrointestinal tract of humans and spread through the fecal-oral route, usually from contaminated food or water. *Y. pseudotuberculosis* is an excellent model organism to study and explore the T3SS as target for therapeutic intervention. The opportunistic pathogen *P. aeruginosa* is the leading cause of hospital-acquired (nosocomial) infections as well as chronic infections in cystic fibrosis patients. In addition, it causes pneumonia and urinary tract, wound, burn, and bloodstream infections. *P. aeruginosa* is a “superbug” with a unique capacity to develop resistance^6^. This is due to a combination of intrinsic, acquired and adaptive resistance. The intrinsic resistance is due to a generally low outer membrane permeability, β-lactamase production and constitutive expression of efflux pumps. Acquired resistance results from horizontal gene transfer and mutations leading to reduced uptake, efflux pump overexpression, target mutations, and expression of antibiotic modifying enzymes such as extended-spectrum β-lactamases. Adaptive resistance is the result of triggering factors such as antibiotics, biocides, polyamines, pH, anaerobiosis, cations, and carbon sources, as well as social behavior in biofilm formation and swarming. These factors modulate expression of genes that lead to increased resistance. This has resulted in multi-drug resistant *P. aeruginosa* strains for which no effective antibiotic treatment is available; moreover, these strains are becoming more frequent.

(-)-Hopeaphenol, a dihydrobenzofuran based resveratrol tetramer, has been isolated from the leaves of the Papua New Guinean rainforest tree *Anisoptera thurifera* in gram quantities^7^. We recently established that this natural product has antibacterial activity towards *Y. pseudotuberculosis, Chlamydia trachomatis* and *P. aeruginosa*^8^. In *Y. pseudotuberculosis* (-)-hopeaphenol irreversibly blocks the T3SS by an unknown mechanism. (-)-Hopeaphenol can be isolated in substantial quantities from natural sources, but in order to establish structure-activity relationships (SARs) and explore the potential for further development, access to analogs is required. However, synthetic efforts toward (-)-hopeaphenol and derivatives have been challenging^9,10^ due to the complex core structure composed of multiple fused rings and the presence of a number of stereocenters. As a first step, we therefore turned our attention to simplified hopeaphenol-related structures and synthesized (dihydro)benzofuran resveratrol dimers, additional stilbenoid natural products and analogues including viniferifuran, ampelopsin A and B, resveratrol-piceatannol hybrid and anigopreissin A^11,12^. Moreover, while (-)-hopeaphenol and related compounds compromise the Lipinski rules of 5^13^ and are at the border of “hard to optimize” structures beyond the rule of 5^14^, the simplified structures of resveratrol dimers and analogues could be more amendable for further exploration.

In this study we tested a set of resveratrol dimers and identified several compounds that block the T3SS in *Y. pseudotuberculosis*. In addition, we developed a new screening method for *P. aeruginosa* by using a green fluorescent protein reporter under the control of the ExoS promoter and confirmed activity against this pathogen as well. Fluorescence microscopy was subsequently used to show the interaction of the T3SS inhibitor viniferifuran with bacterial cells.

## Results

In this study, we investigated the biological effects of selected natural benzofuran resveratrol dimers and analogues on the T3SS in comparison to (-)-hopeaphenol. These compounds are readily prepared by biomimetic methods or total synthesis and include ε-viniferin, ω-viniferin, ampelopsin B, ampelopsin A, viniferifuran, dehydroampelopsin B, δ-viniferin, dehydro-δ-viniferin, anigopreissin A and a resveratrol-piceatannol hybrid (Table 1, see Methods for details).

**Table 1:** Activity against the T3SS and bacterial growth of *Y. pseudotuberculosis* (see Methods for details).

Structure	No	Name	E-lux IC_50_ µM	YopH IC_50_ µM	Growth inhibition
	1	(-)-Hopeaphenol	10	7	No
	2	(±)-Ampelopsin A	>50	>50	No
	3	(±)-Ampelopsin B	>50	40	No
	4	(±)-Dehydro-ampelopsin B	40	20	No
	5	(+)-ε-Viniferin	>50	15	No
	6	(±)-ε-Viniferin	>50	30	No
	7	(±)-Cis-ε-viniferin (Omega-viniferin)	>50	48	No
	8	Viniferifuran	10	4	No
	9	(±)-δ-Viniferin	29	15	No
	10	Dehydro-δ-viniferin	5	4	ND*
	11	Anigopreissin A	12	6	Yes
	12	Resveratrol-piceatannol hybrid	15	8	ND*
	13	Propagylated viniferifuran	6	3	ND*

*Not determined.

### Inhibition of *yopE* expression and YopH secretion

**Figure 1:**
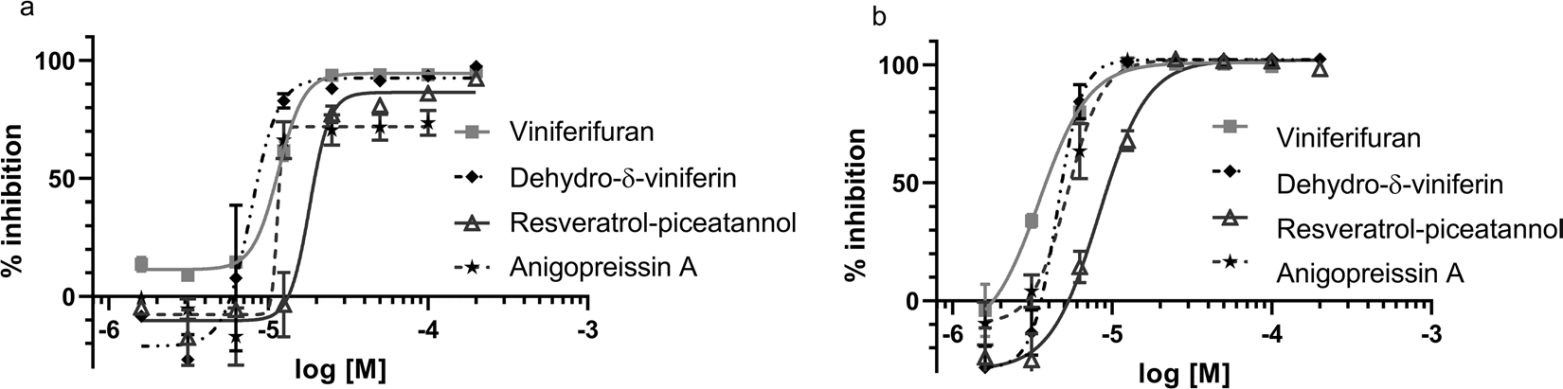
Viniferifuran (8), dehydro-δ-viniferin (10), anigopreissin A (11) and resveratrol-piceatannol hybrid (12) show dose-dependent inhibition of *yopE* expression (**a**) and YopH secretion (**b**). The YPIII(pIB102E-lux) was induced for T3S and compounds were added at 1–100 µM. The graphs show the mean value (n = 3) +/−SD.

The compounds were tested for inhibition of the T3SS in the combined *yopE-lux* and YopH phosphatase assay for dose-dependent activity as described previously^8^. In addition, inhibition of bacterial growth was measured to allow identification of T3SS selective inhibitors with little or no effect on bacterial viability. The results are compiled in Table 1. The direct half of (-)-hopeaphenol (1) i.e. ampelopsin A (2) and B (3) as well as dehydroampelopsin B (4), which all contain a central 7-membered ring structure, showed no or modest inhibition of the T3SS. Similar data was obtained for the related opened form compounds ε-viniferin (5) and ω-viniferin (7) (IC_50_> 50 μM, *yopE*). The related benzofuran based viniferifuran (8), on the other hand, showed good inhibition of the T3SS (Fig. 1) with an IC_50_ of 10 μM (*yopE*) and 4 μM (YopH), without any effect on bacterial growth, i.e. the inhibition profile is similar to that of the original lead (-)-hopeaphenol. The fact that the inhibition profile of the resveratrol tetramer (-)-hopeaphenol is maintained by the resveratrol dimer viniferifuran is encouraging since it has a simplified structure that can be prepared in gram quantities in only four steps^11^. The resveratrol-piceatannol hybrid (10) that differs from viniferifuran in its hydroxylation pattern has a similar inhibition profile as viniferifuran *in vitro*. δ-Viniferin (9) with the stilbene substitution at position 6 displayed a moderate activity (IC_50_ = 29 μM, *yopE*) and the corresponding benzofuran analog dehydro-δ-viniferin (10) was found to be equally potent as viniferifuran. Anigopreissin A (11), with the stilbene group in position 7, inhibits the T3SS, but its detrimental effect on bacterial growth indicates that the inhibition is non-selective (Fig. S1, Table 1). All compounds with a benzofuran core structure i.e. viniferifuran, dehydroampelopsin B, anigopreissin A, dehydro-δ-viniferin and the resveratrol-piceatannol hybrid displayed significant to moderate activities (IC_50_ = 5–40 μM, *yopE*).

### Viniferifuran is an irreversible inhibitor of T3SS

**Figure 2:**
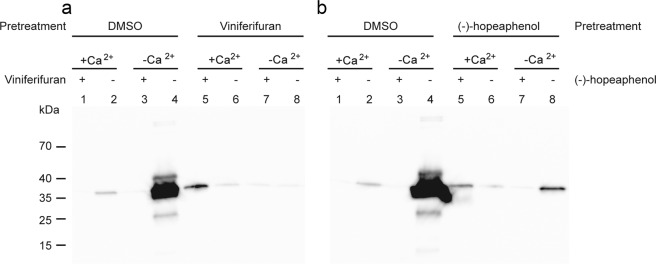
The effect of viniferifuran (**a**) and (-)-hopeaphenol (**b**) on *Y. pseudotuberculosis* is irreversible. Western blot analysis of the reversibility of viniferifuran and (-)-hopeaphenol treatment of *Y. pseudotuberculosis*. Lanes 1–4 show the secretion profile of bacteria pretreated with DMSO and lanes 5–8 bacteria pretreated with viniferifuran or (-)-hopeaphenol. After pretreatment the two cultures were divided into four, washed and two of them were diluted in lysogeny broth (LB) supplemented with Ca^2+^ (non-inducing conditions) and the other two were diluted in Ca^2+^ depleted LB (inducing conditions). (-)-Hopeaphenol, viniferifuran (40 µM final concentration) or DMSO were then added and subsequetly the protein secretion profiles were analysed.

Viniferifuran was further tested for reversibility using a Western blot analysis as described previously^8^. Vinferifuran (8) and (-)-hopeaphenol (1) were added to the bacteria and after induction of T3SS, the compounds were washed away and the bacterial solution was divided into two, where one was treated again with compound under T3SS inducing conditions (-Ca^2+^) and one was used as a control by adding dimethyl sulfoxide (DMSO) under inducing conditions. Both compounds irreversibly block secretion of effector molecules, i.e. the T3SS could not be reactivated by washing (Fig. 2).

### Binding of viniferifuran to *Yersinia pseudotuberculosis*

**Figure 3:**
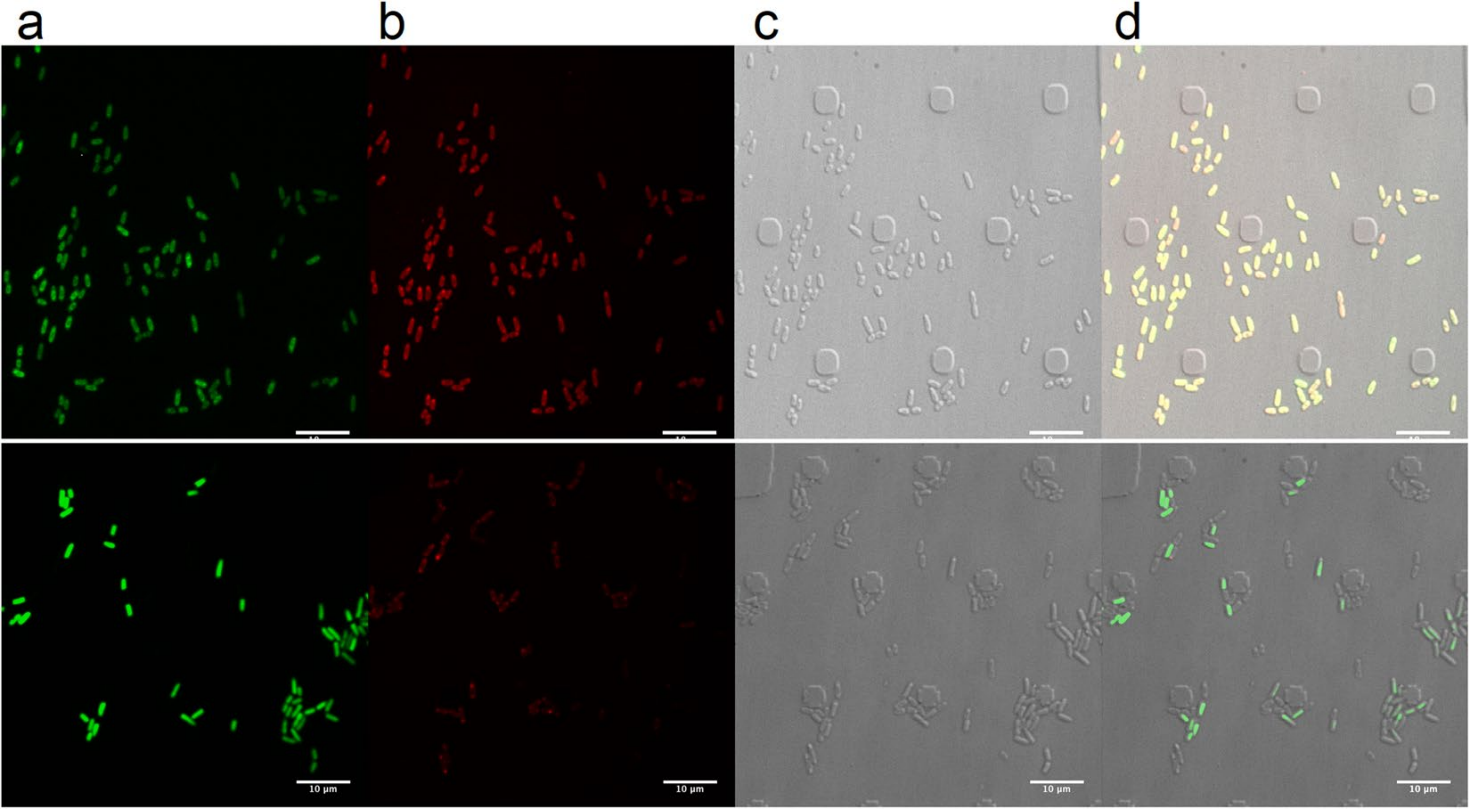
Binding of viniferifuran to bacteria. Top panel shows the YPIII(pIB102, pFU95gfp) strain treated with 10 µM compound 13 followed click chemistry with sulfo-cyanine azide. Bottom panel shows bacteria treated with 10 µM compound 13 and 50 µM viniferifuran followed click chemistry with sulfo-cyanine azide. (**a**) GFP signal from the bacteria, (**b**) red signal, showing bacteria labeled with compound 13, (**c**) all bacteria visible with light microscopy, (**d**) merged pictures.

Propagylation of viniferifuran with propargyl bromide resulted in three mono-propargylated compounds that could be separated by chromatography (Supplementary information). These fractions were tested for their biological activity and compound 13 was selected for further experiments due to its retained biological activity (Table 1) as well as the fact that it could be produced in the highest amounts of all of the mono-propargylated products. *Y. pseudotuberculosis* (YPIII (pIB102, pFU95gfp)), which constitutively expresses green fluorescent protein (GFP), was treated with compound 13 or DMSO followed by cupper catalyzed click chemistry of the propargyl functionality with the azide group of sulfo-cyanine azide. The bacteria were analyzed using confocal spinning disc microscopy (The CellASIC ONIX2 Microfluidic System, Merck). All bacteria display green fluorescence from the constitutively expressed GFP and the bacteria that has bound propagylated viniferifuran labeled with the azide fluorophore appear red. The results showed a clear binding of viniferifuran to the bacteria (Fig. 3b in top panel). Competition experiments showed that the binding could be outcompeted by adding five times more of free viniferifuran compared to the propargylated compound 13 (Fig. 3). Furthermore, the results showed that the compound binds directly to the bacteria without causing aggregation of the bacteria.

### Inhibition of *exoS* expression in *P. aeruginosa*

**Figure 4:**
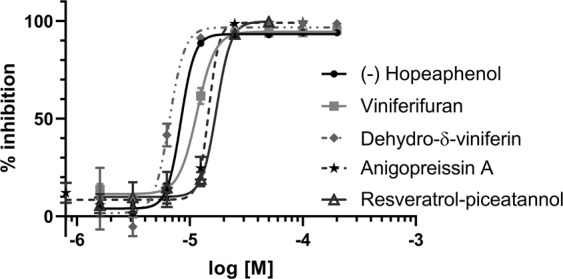
Inhibition of ExoS expression by (-)-hopeaphenol (1), viniferifuran (8), dehydro-δ-viniferin (10), anigopreissin A (11) and resveratrol-piceatannol hybrid (12). Wild-type *P. aeruginosa* PAK(pCS500) with GFP under the control of the *exoS* promoter was treated with different concentration of compounds under T3SS-inducing contitions. As controls the the untreated PAK(pCS500) (wt) and PAK*exsA*(pCS500) (*exsA*^−^) were used. The expression of *exoS* was calculated as percenteage of the untreated wild-type PAK(pCS500). Samples were run in triplicates and the experiment was repeated three times. The graph shows the mean value (n = 3) +/−SD.

The compounds were further tested for efficacy on the opportunistic pathogen *P. aeruginosa*. A reporter-gene was constructed where the promoter of the *P. aeruginosa* T3S-toxin, ExoS, was transcriptionally fused to the GFP reporter gene. The gene construct was then cloned into the high copy plasmid pUCP18, together with *orf1*, coding the chaperon of ExoS^15^. To be able to select for the plasmid, the gene coding for gentamicin resistant was inserted into the plasmid, creating plasmid pCS500 (Supporting information). The resulting plasmid was inserted into wild-type *P. aeruginosa* strain PAK and the T3SS activator mutant PAK*exsA*, resulting in strains that expresses the GFP when the ExoS promoter is activated. The T3SS is induced by incubating the bacteria in calcium depleted medium at 37 °C. The wild-type PAK and the PAK*exsA* strains with the reporter plasmid were grown under induced conditions and the compounds were added to the growth medium at different concentrations. After 16 h of incubation the GFP signal was measured in a plate reader (Biotek Synergy) and the inhibition of ExoS expression was calculated. The PAK(pCS500) control (no compound added) showed a high GFP level corresponding to a high expression of *exoS*, while the PAK*exsA*(pCS500) control strain showed a low level of GFP corresponding to a low level of *exoS* expression. The level of GFP seen by the PAK*exsA*(pCS500) strain was equal to a full inhibition of *exoS* by a fully active compound (Figure S3). Simultaneously, the inhibition of bacterial growth was measured to select for selective T3SS inhibitors. All compounds with a benzofuran core structure i.e. viniferifuran (8), dehydroampelopsin B (4), anigopreissin A (11), dehydro-δ-viniferin (10) and the resveratrol-piceatannol hybrid (12) exhibited significant activities by inhibiting expression of ExoS in a dose dependent manner, with IC_50_ values of approximately 8–30 µM (Fig. 4 and Table 2). However, compounds containing the central 7-membered ring structure displayed higher IC_50_ values, as exemplified by comparison of dehydroampelosin B (4, IC_50_ 33 µM) and viniferifuran (8, IC_50_ 11 µM) (Table 2). None of the compounds showed inhibition of bacterial growth and could therefore be considered as selective T3SS inhibitors in *P. aeruginosa*.

**Table 2:** Summery of efficacy and toxicity towards *P. aeruginosa* and eukaryotic cells (J774).

No	Name	IC_50_ ExoS-gfp	Tocxicity P.a.	Toxicity* J774	Efficacy Pa J774
1	(-)-Hopeaphenol	9	No	No	Yes
2	(±)-Ampelopsin A	>200	No	No	No
3	(±)-Ampelopsin B	134	No	No	No
4	(±)-Dehydro-ampelopsin B	33	No	No	No
6	(±)-ε-Viniferin	89	No	No	No
8	Viniferifuran	11	No	Tox	Moderate
10	Dehydro-δ-viniferin	8	No	Tox	Yes
11	Anigopreissin A	15	No	Some tox	No
12	Resveratrol-piceatannol hybrid	14	No	Some tox	Yes

*For more information about toxicity on J774 cells, see Fig. S2.

### Inhibition of T3S-mediated cytotoxicity of eukaryotic cells

**Figure 5:**
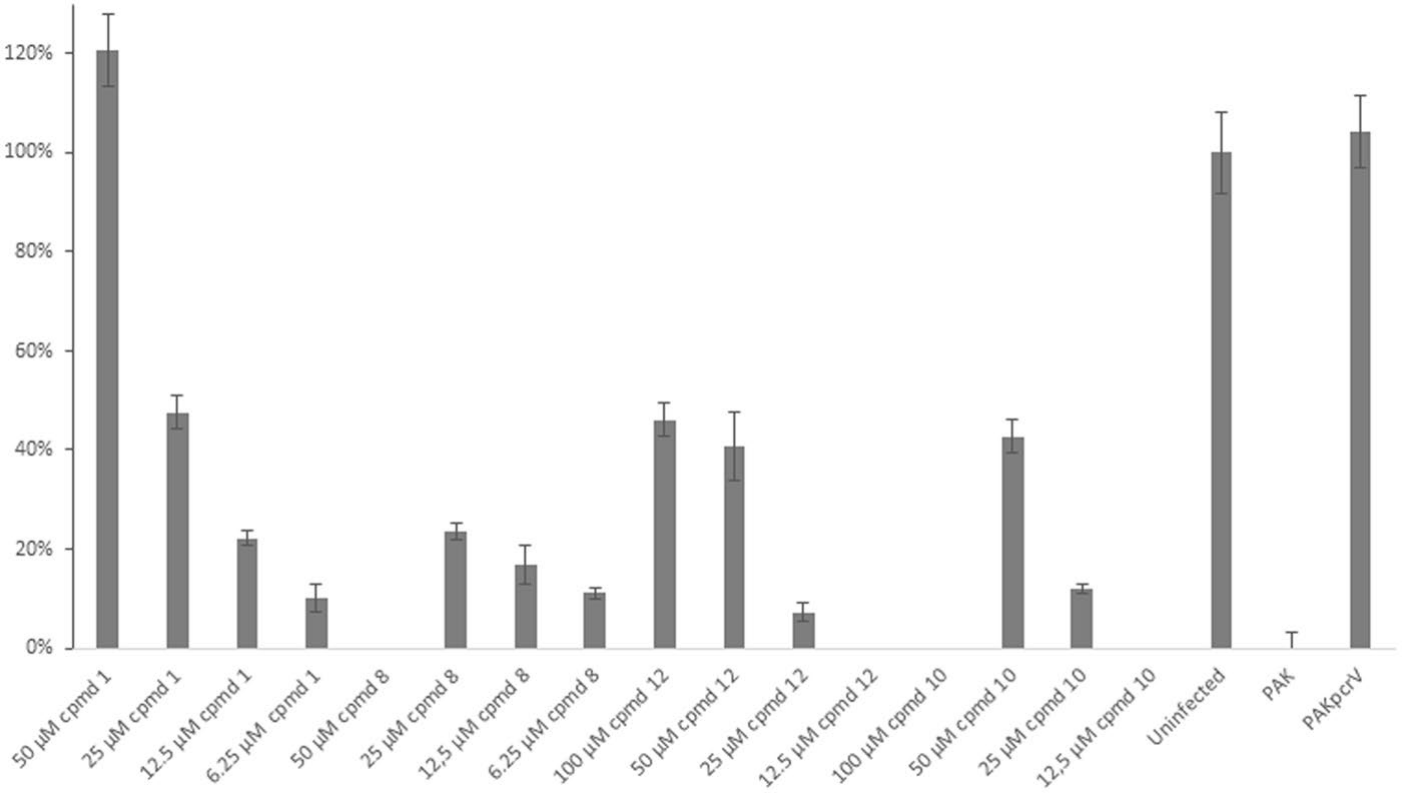
Effect of the (-)-hopeaphenol analogues on *P. aeruginosa* infection. J774 cells were infected with wild-type *P. aeruginosa* strain PAK and compounds were added and the T3SS dependent cell survival was analysed using a viability staining method. As controls uninfected cells, cells infected with the PAK*pcrV*-mutant and cells infected with the wild-type strain (PAK) were used. The survival was calculated as percenteage of the uninfected control. Samples were run in triplicates and the experiment were repeated three times. Viniferifuran (8) and dehydro-δ-viniferin (10) were very toxic at 100 µM, while some toxicity could be seen for resveratrol-pinceatannol hybrid (12). The graph shows the mean value (n = 3) +/−SD after 6 h of infection.

The T3SS is important for *P. aeruginosa* infection of eukaryotic cells. A T3SS activator mutant or a mutant lacking the T3SS tip protein PcrV is not able to infect eukaryotic cells^16,17^. The J774 cells (ATCC) were infected with the wild-type PAK strain and the T3SS mutant with or without compounds. The T3SS mediated cytotoxic effect on the eukaryotic cells was measured by using a viability staining method, Uptiblue (Interchim), after 4, 5 and 6 h of infection. Simultaneously, uninfected cells were treated with compounds and toxicity towards eukaryotic cells was determined using the same assay. The cells were also studied by microscopy after 6 h to assess cell morphology. The results showed a clear dose-dependent rescue effect of (-)-hopeaphenol (1), with complete inhibition of cytotoxicity at 50 µM, which is in agreement with previous results^8^ (Fig. 5). Additionally, dehydro-δ-viniferin (10) and resveratrol-pinceatannol hybrid (12) showed a significant dose-dependent rescue effect on eukaryotic cells, while viniferifuran (8), (±)-ε-viniferin (5) and anigopreissin A (11) showed modest or no efficacy (Fig. 5). Viniferifuran (8) and dehydro-δ-viniferin (10) showed substantial toxicity towards eukaryotic cells at 100 µM, while some toxicity could be seen for the resveratrol-pinceatannol hybrid (12, Figure S2). Toxicity towards eukaryotic cells limits the possibility to study and accurately assess virulence blocking properties of these compounds.

## Discussion

Most antibiotics in clinical use today are either natural products of microbial origin or improved derivatives obtained by synthesis. Development of new antibiotics based on new synthetic scaffolds have proved to be challenging and this holds true also for identification of chemical entities that block virulence systems by mechanisms distinct from conventional antibiotics e.g. attenuation of T3SS in Gram-negative pathogens. A substantial number of synthetic T3SS inhibitors have been identified by predominantly phenotypic screening, and a selection of compounds have entered medicinal chemistry programs as summarized in recent review articles^18,19^. Several studies indicate that natural sources also have the potential to provide T3SS inhibitors for further exploration. Screening of marine invertebrate extracts led to the identification of the glycolipid caminoside A as an inhibitor of T3SS in *enteropathogenic Eschericia coli* (EPEC)^20^ and later caminocide B-D were also found to be T3SS inhibitors^21^. In addition to activity against T3SS in EPEC, the caminosides also display antimicrobial activity against vancomycin resistant *Enterococcus* and methicillin resistant *Staphylococcus aureus*, suggesting that these compounds act as general antibiotics rather than selective T3SS inhibitors^20,21^. In another study guadinimine A-F, isolated from a soil-derived *Streptomyces* broth, were found to block T3SS dependent hemolysis of erythrocytes by EPEC^22,23^. Aurodox is a known actinomycete derived antibiotic that inhibits protein biosynthesis by binding to bacterial elongation factor Tu^22,23^. Recently it was reported that aurodox inhibited EPEC T3SS-mediated hemolysis and protected mice infected with a lethal dose of *Citrobacter rodentium*^24^. The mechanism for its T3SS inhibition has however not been established. Pseudoceramines A-D and spermatinamine produced by the marine sponge *Pseudoceratina* spp. were identified as putative inhibitors of T3SS in *Y. pseudotuberculosis*, but the compounds also exhibited a detrimental effect on bacterial growth, indicating general antibacterial properties^25^. Phenolic compounds involved in plant defense signaling have been reported to block the T3SS in *P. aeruginosa* via the GacS-GacA two-component signal transduction system^26^. Cytosporone B, a fungal metabolite, and analogues selectively block the secretion of *Salmonella* pathogenicity island 1 (SPI-1)-associated effector proteins, without significant toxicity against bacteria^27^ and similar effects have been reported for prenylated plant flavonoids, of which licoflavonol proved to be the most promising compound^28^. It was recently reported that the T3SS of *Y. pseudotuberculosis* is effectively blocked by piericidin A1, a metabolite produced by a marine actinobacterium^29^. The compound was identified by screening of a library composed of marine-derived extracts and its virulence blocking activity is not attributed to general toxicity towards the bacteria.

Based on our finding that the plant derived complex resveratrol tetramer (-)-hopeaphenol (1) is an irreversible T3SS inhibitor we tested a series of resveratrol dimers that can be obtained by biomimetic or total synthesis. We identified the resveratrol dimer viniferifuran (8) and analogues as inhibitors of T3SS that target both *Y. pseudotuberculosis* and *P. aeruginosa*. Since we could observe inhibition of T3SS-toxins in both *Y. pseudotuberculosis* and *P. aeruginosa* it is not likely that the resveratrol dimers act on the toxins per se, since they are different between the bacterial species. The compounds rather act on a homologous function of the T3SS as the needle like structure or tip-proteins on the bacterial surface or some common regulatory system. Viniferifuran was found to irreversibly block the T3SS, indicating that (-)-hopeaphenol and viniferifuran might act by the same mechanism, although the chemical structures are substantially different. The efficacy of viniferifuran in cell infection experiments could not be accurately assessed due to its toxicity toward J774 cells.

Due to its ease of preparation, we still consider viniferifuran a promising scaffold amendable for medicinal chemistry to optimize its potency toward diverse Gram-negative pathogens and reduce its toxicity. The resveratrol-piceatannol hybrid (12) and the dehydro-δ-viniferin (10) showed better efficacy to rescue eukaryotic cells from infection, however they both show toxicity towards the eukaryotic cells at high concentrations, even though the toxicity of the resveratrol-piceatannol hybrid is minor compared to viniferifuran. (-)-Hopeaphenol is non-toxic toward both bacteria and eukaryotic cells and possibly this can be attributed to its size, which likely prevents cell permeability and suggest that (-)-hopeaphenol acts on the bacterial surface.

Incubation of *Y. pseudotuberculosis* with the monopropargylated viniferifuran derivative 13, followed by click chemistry using sulfo-cyanine azide and subsequent confocal spinning disc microscopy, suggested that viniferifuran might be located at the outer membrane of the bacteria but an intracellular localization cannot be excluded. Importantly, the pictures showed that the compound binds to the bacteria and the T3SS inhibition is not due to e.g. aggregation of bacteria.

Interestingly, all the most promising compounds in the present study share the unsaturated benzofuran scaffold as opposed to parent compound (-)-hopeaphenol that contains the related dihydrobenzofuran scaffold. Other compounds with the dihydrobenzofuran core e.g. ampelopsin A (2) and B (3) were essentially void of activity.

In conclusion, we have identified resveratrol dimers as novel inhibitors of T3SS in *Y. pseudotuberculosis* and *P. aeruginosa*. Compared to the parent compound, (-)-hopeaphenol, these compounds are amendable to medicinal chemistry and are thus useful as starting points to develop lead compounds and initiate drug discovery programs.

## Methods

### Compounds

(-)-Hopeaphenol (1) was obtained from extraction of *Anisoptera thurifera* leafs as described previously^7^ and (+)-ε-viniferin (5) was from Sigma Aldrich. (±)-ε-Viniferin (6), (±)-ω-viniferin (7), (±)-ampelopsin B (3), viniferifuran (8), (±)-dehydroampelopsin B (4) were prepared following a biomimetic or total synthetic procedures^11,12^. (±)-Ampelopsin A (2) was prepared from ε-viniferin following a biomimetic procedure^30^. Anigopreissin A (11) was isolated from the Australian plant *Macropidia fuliginosa*^31^ or prepared as reported previously^11^. Dehydro-δ-viniferin (10) and resveratrol-piceatannol hybrid (11) were prepared as described previously^11^.

### *yopE* reporter gene and YopH phosphatase assays

The combined luciferase and phosphatase assay was performed essentially as previously described^8^. The *Y. pseudotuberculosis* serotype III strain YPIII(pIB102E-lux) (Table 3) was grown overnight in Luria broth (LB) supplemented with chloramphenicol, diluted to an OD_600_ of 0.08 in calcium depleted LB medium (addition of ethyleneglycol- bis(β-aminoethyl)-N,N,Nʹ,Nʹ-tetraacetic acid (EGTA) and MgCl_2_, at a final concentration of 5 mM and 20 mM, respectively) and incubated in 96-well plate 100 µl/well (Nunc flat bottom, white). The compounds were added to a final concentration between 1 and 100 µM. The plate was incubated for 1 h at 26 °C and 2 h at 37 °C, shaking. After incubation 10 µl of the bacterial suspension was transformed to a new 96-well plate (Nunc flat bottom, transparent) containing 90 µl of phosphatase mixture (25 mM *p*-nitro phenyl phosphatase, 40 mM 2-(N-morpholino) ethanesulfonic acid, pH 5.0, and 1.6 mM dithiotheitol in water). The plate was incubated for 15–20 min at 37 °C. 20 µl 1 M NaOH was added to stop the reaction and the absorbance, i.e. YopH secretion, was measured at 405 nm in a plate reader (Wallac Viktor^2^ 1420 Multilabel counter). The remaining 90 µl of bacterial suspension in the white 96-well plate was used for measuring the luciferase activity, i.e. the *yopE* expression), by adding 50 µl of decanal in water (5 µl/500 ml) and the chemiluminescence was detected in a microplate reader (Wallac Viktor^2^ 1420 Multilabel counter). The IC_50_ values were calculated using GraphPad Prism software.

**Table 3:** Characteristics and references of bacterial strains and crusal plasmids used in this study.

Strain or plasmid	Characteristics and genotype	Reference/source
***Yersinia pseudotuberculosis***		
YPIII(pIB102)	*yadA*	^32^
YPIII(pIB102-Elux)	Luciferace reporter under the control of *yopE* promoter	^33^
YPIII(pIB102; pFU95)	Constitutive *gfp* expression	This paper
***Pseudomonas aeruginosa***		
PAK	Wild-type	FOI*
PAK*pcrV*	*pcrV*	^17^
PAK*exsA*	*exsA*	^34^
PAK(pCS500)	*gfp* under the control of the *exoS* promoter	This paper
PAK*exsA*(pCS500)	*exsA*, *gfp* under the control of the exoS promoter	This paper
**Relevant plasmids**		
pFU95	Constitutive *gfp* expression	^35^
pTS103	Source of *Orf1* and *exoS*	^15^
pCS500	*gfp* under the control of the exoS promoter, Gm resistant	This paper
pRIC-gfp	Source of *gfp*	FOI*
p34S-Gm	Ap^R^ Gm^R^, source of Gm^R^ cassette	^36^

*Swedish Defence Research Agency.

### Inhibition of bacterial growth

For growth control, the *Y. pseudotuberculosis* serotype III strain YPIII(pIB102E-lux) (Table 3) was grown overnight in LB supplemented with chloramphenicol, diluted to an OD_600_ of 0.2 in LB medium with 2.5 mM CaCl_2_ and added into 96-well plate 100 µl/well (Nunc flat bottom, transparent). Compounds were then added and the plate was incubated at 37 °C for 24 h, shaking. The absorbance at OD_600_ was measured every hour for 8 h and then after 24 h in a microplate reader (Wallac Viktor^2^ 1420 Multilabel counter).

### Western blot analysis of supernatant samples of *Y. pseudotuberculosis* for studying reversibility

The experiment was performed essentially as described earlier^8^. A final concentration of 40 µM of the compounds were added to a 1:20 diluted overnight culture of YPIII(pIB102). Control cultures received only DMSO. The cultures were incubated at 26 °C for 30 min followed by 2 h at 37 °C to induce T3SS. The samples were then centrifuged and the pellet washed once with LB where after it was suspended in LB complemented with 20 mM MgCl_2_ and 5 mM EGTA for Ca^2+^ depletion (induction of T3SS) or 2.5 mM CaCl_2_. The samples were divided and 40 µM compound or DMSO was added followed by incubation for 45 min at 37 °C. The cultures supernatant was mixed with sample buffer and loaded on to a 12% sodium dodecyl sulfate (SDS) polyacrylamide gel and blotted onto a PVDF membrane. Polyclonal rabbit anti Yop-antiserum and polyclonal goat anti-rabbit immunoglobulin conjugated with horseradish peroxidase (HRP) (Dako Denmark) were used together with Millipore Immobilon Western chemiluminescent HRP substrate to determine Yop proteins in the supernatant. The chemiluminiscense signals were detected with a charge-coupled device (CCD) camera.

### Visualizing viniferifuran binding to bacterial cells

The *Y. pseudotuberculosis* serotype III strain YPIII (pIB102; pFU95) (Table 3) was grown overnight in LB supplemented with carbenicillin. 100 µl YPIII (pIB102; pFU95) without compound in calcium depleted LB medium at an OD_600_ of 0.11, was mixed with propagylated viniferifuran (13) to a final concentration of 10 µM. To investigate selectivity of the compound viniferifuran (8) was added to a final concentration of 50 µM together with propagylated viniferifuran (13). The DMSO concentration was kept to 1% in all samples and bacteria treated only with 1% DMSO were used as control. The samples were incubated in 96-well plates for 1 h at 26 °C and 2 h in 37 °C with shaking. After incubation, the bacteria were diluted 1/5 in phosphate buffered saline (PBS) and 50 µl was loaded into a CellASIC ONIX B04A-03 Microfluidic Bacteria Plate of the CellASIC ONIX2 Microfluidic System (Merck), with a flow rate of 15 s at 4 psi twice to get them into the plate layers. After additional 5–10 times at 6 psi the bacteria reached the correct position (trap heights of 0.7 µm).

The click chemistry reaction mixture was made with a final concentration of CuSO_4_ (0.1 mM), tris[(1-benzyl-1*H*-1,2,3-triazol-4-yl)methyl]amine (TBTA, 128 µM), sodium ascorbate (5 mM), sulfo-cyanine5 azide (100 µM, Lumiprobe, ex/em 646/662) in PBS. The plate was washed with for PBS 10 min at 4 psi, where after the click reaction mix was added for 5 min at 8 psi and 60 min at 4 psi. Finally, the plate was washed with PBS for 10 min at 8 psi and 20 min at 4 psi and the results were analyzed using a Confocal spinning disc microscope, far red 647 laser. The plate was incubated at 37 °C during the experiment.

### Cloning and *exoS* expression assay

The pRIC-GFP plasmid was cut with PstI and EcoRI and the GFP fragment was purified and ligated into pTS103 plasmid^15^ cut with NsiI and EcoRI. The resulting plasmid was purified and cut with BamHI and EcoRI and ligated into the pUCP18 plasmid cut with BamHI and EcoRI. The resulting plasmid was cut with ScaI (blunt, cutting in the amp gene) and the gentamicin (Gm) gene was inserted by cleaving the p34S-Gm plasmid with Ecl136II (SstI) (blunt). The resulting plasmid, pCS500, was resistant to gentamicin and the *gfp*-gene is transcriptionally fused with the *exoS* promoter. pCS500 was transformed into the *P. aeruginosa* strain PAK and the T3SS activator mutant PAK*exsA*.

Overnight cultures of PAK(pCS500) and PAK*exsA*(pCS500) were diluted 1:30 in LB supplemented with 3 µg/ml Gm and incubated at 37 °C for 6 h on a rotary shaker. The cultures were then diluted to an OD_600_ of 0.0004 in calcium deleted LB supplemented with 3 µg/ml Gm and added into a flat-bottom 96-well transparent plate (Nunc). Compounds were added to give a final concentration of 1–100 µM. As a positive control 1% DMSO was added to the PAK(pCS500) strain and as a negative control 1% DMSO was added to the PAK*exsA* (pCS500). The fluorescence was measured after 16 h of incubation on a rotary shaker (ex/em, 488/509) in a plate reader (Biotek Synergy). The value for the wt PAK(pCS500) strain with no additives was set to 100% GFP expression (i.e. 100% ExoS expression). The IC_50_ values were calculated using GraphPad Prism software. Inhibition of bacterial growth was also measured after 16 h using the same plate by measurement of the absorbance at OD_600_. All experiments were carried out in triplicates.

### Infection and toxicity assay

J774 cells (ATCC) was seeded to a concentration of 5 × 10^4^ cells/well (100 µl/well) in 96-well plates and incubated 18 h prior to infection in Dulbecco’s modified eagle medium (DMEM, GlutaMAX (Glu), 3 µg/ml Gentamicin (Gm) + 10% FCS). Overnight cultures of PAK and PAK*pcrV* were diluted 1:10 in DMEM (Glu) medium and incubate at 37 °C, 250 rpm for 1 h. Meanwhile, the cells were wash once with PBS pH 7.2, where after 30 µl of compound solutions was added diluted in DMEM (Glu, 10% FCS). 1% DMSO was added to the control wells. 30 µl of the induced PAK bacteria solution, OD_600_ = 0.0008, was then added to the wells giving a final OD_600_ of 0.0004. PAK*pcrV* mutant, deficient for translocation of exoenzymes, was used to control that the effect on the cells was T3SS dependent. In a parallel plate, the compound solutions were added without bacteria to screen for toxicity. A viability staining method to investigate cell survival, after 2.5 h, 10 µl Uptiblue (Interchim, France) was added to each well, efficacy and toxicity of the compounds was measured 4, 5 and 6 h after infection (ex/em, 535/595) in a plate reader (Biotek Synergy). For efficacy, the compounds were tested at 50, 25, 12.5 and 6.25 µM in triplicates.

## Supplememtary information

Supplememtary information.
