# Supplementary material for: Exploring resveratrol dimers as virulence blocking agents – Attenuation of type III secretion in Yersinia pseudotuberculosis and Pseudomonas aeruginosa
Source: Sci Rep. 2020 Feb 7;10:2103. doi: 10.1038/s41598-020-58872-0 (PMC7005745; doi:10.1038/s41598-020-58872-0)
Supplement: Supplementary file 1 — Supplememtary information. [file 41598_2020_58872_MOESM1_ESM.pdf]

Exploring resveratrol dimers as virulence blocking agents – Attenuation of type III secretion in *Yersinia pseudotuberculosis* and *Pseudomonas aeruginosa*

Charlotta Sundin[a]*, Caroline E. Zetterström[a], Duc Duy Vo[a], Robert Brkljača[b], Sylvia Urban[b] and Mikael Elofsson[a]

[a] Department of Chemistry Umeå University, SE 901 87 Umeå, Sweden

[b] School of Science (Applied Chemistry and Environmental Science), RMIT University, GPO Box 2476 Melbourne, Victoria, 3001, Australia

e-mail address corresponding author: charlotta.sundin@umu.se

Sequence pCS500

>pCS500 Orf1, ExoS promoter, GFP 1682bp

TAGAGGATCCTCCTTTCGCCCGACTGGGCTCAGCGTAGCTCTTCGGCGGCGGCGACCAGTTGTTCG
AGTTGGTGGTGGATCTGGGCCCTGTCCAGCAACTGCAAGGGCTGGCGGTTCCAGAGCAGCCGTTC
GCCGCTTTCGGGGTCACGGCCCAGGATCGGCTTGCAAGGGTCCTGGCTGAACAGGTTCTGCTCGTT
GGCCGTAGCGTCTCCTTGTCCTTCCAGGCGGGTGAACATCAACAGGTGATCGGTCGGATGTTCTGC
CAGATGGCAGAGATGCGGGCCCACCTGCAGGCTGAGTACGCTCTCCTCGTCGTTGGGCGTCGGGA
GATCGAGAGCGAGAAAAAGCTGGTGGATGGCGGCGCGGTAGAGTGGATTCATGGCGTGTTCCGA
GTCACTGGAGGCAGCCATTAGAGCAGTGCCAGCCCGGAGAGACTGTTAATCGTGGTTCTCTTTTTTT
AGGTTTTGCCGCTGCCGATTCCAGTGAAAAAACGGCGGCCAATCCTGATAGGCGATGGGGTTTCCC
GTTCCTAGACTGGCGGAGAAACATCAGGAGAAGGCAACCATC**ATG**CAGATTTAAGAAGGAGATATA
CATATGAGTAAAGGAGAAGAACTTTTCACTGGAGTTGTCCCAATTCTTGTTGAATTAGATGGTGATG
TTAATGGGCACAAATTTTCTGTCAGTGGAGAGGGTGAAGGTGATGCAACATACGGAAAACTTACCC
TTAAATTTATTTGCACTACTGGAAAACTACCTGTTCCATGGCCAACACTTGTCACTACTTTCGCGTAT
GGTCTTCAATGCTTTGCGAGATACCCAGATCATATGAAACAGCATGACTTTTTCAAGAGTGCCATGC
CCGAAGGTTATGTACAGGAAAGAACTATATTTTTCAAAGATGACGGGAACTACAAGACACGTGCTG
AAGTCAAGTTTGAAGGTGATGCCCTTGTTAATAGAATCGAGTTAAAAGGTATTGATTTTAAAGAAGA
TGGAAACATTCTTGGACACAAATTGGAATACAACTATAACTCACACAATGTATACATCATGGCAGAC
AAACAAAAGAATGGAATCAAAGTTAACTTCAAAATTAGACACAACATTGAAGATGGAAGCGTTCAAC
TAGCAGACCATTATCAACAAAATACTCCAATTGGCGATGGCCCTGTCCTTTTACCAGACAACCATTAC
CTGTCCACACAATCTGCCCTTTCGAAAGATCCCAACGAAAAGAGAGACCACATGGTCCTTCTTGAGTT
TGTAACAGCTGCTGGGATTACACATGGCATGGATGAGCTCTACAAATAGAATTCGTAATCATGTCAT
AGCTGTTTCCTGTGTGAAATTGTTATCCGCTCACAATTCCACACAACATACGAGCCGGAAGCATAAA
GTGTAAAGCCTGGGGTGCCTAATGAGTGAGCTAACTCACATTAATTGCGTTGCGCTCACTGCCCGCT
TTCCAGTCGGGAAACCTGTCGTGCCAGCTGCATTAATGAATCGGCCAACGCGCGGGGAGAGGCGG
TTTGCGTATTGGGCGCTCTTCCGCTTCCTCGCTCACTGACTCGCTGCGCTCGGTCGTTCGGCTGCGG
CGAGCGGTATCAGCTCACTCAAAGGCGGTAATACGGTTATCCACAGAATCAGGGGATAACGCAGGA
AAGAACATGTGAGCAAAAGGC

Orf1 gene reverse transcription

AACGGCGGCCAATCCTGATA ExoSf21 primer

ATGCAG NsiI PstI site after ligation

ATG starting codon of ExoS

GFP gene

GGATCC BamHI site

GAATTC EcoRI site

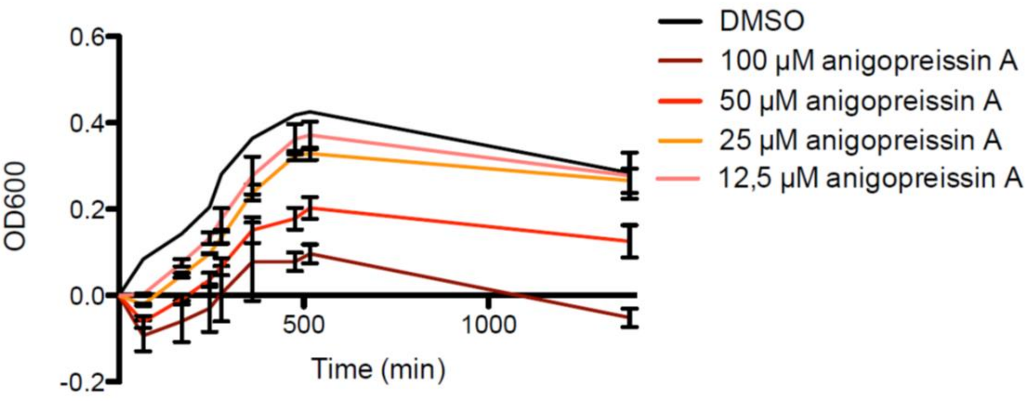

Figure S1. Inhibition of *Y. pseudotuberculosis* growth by anigopreissin A.

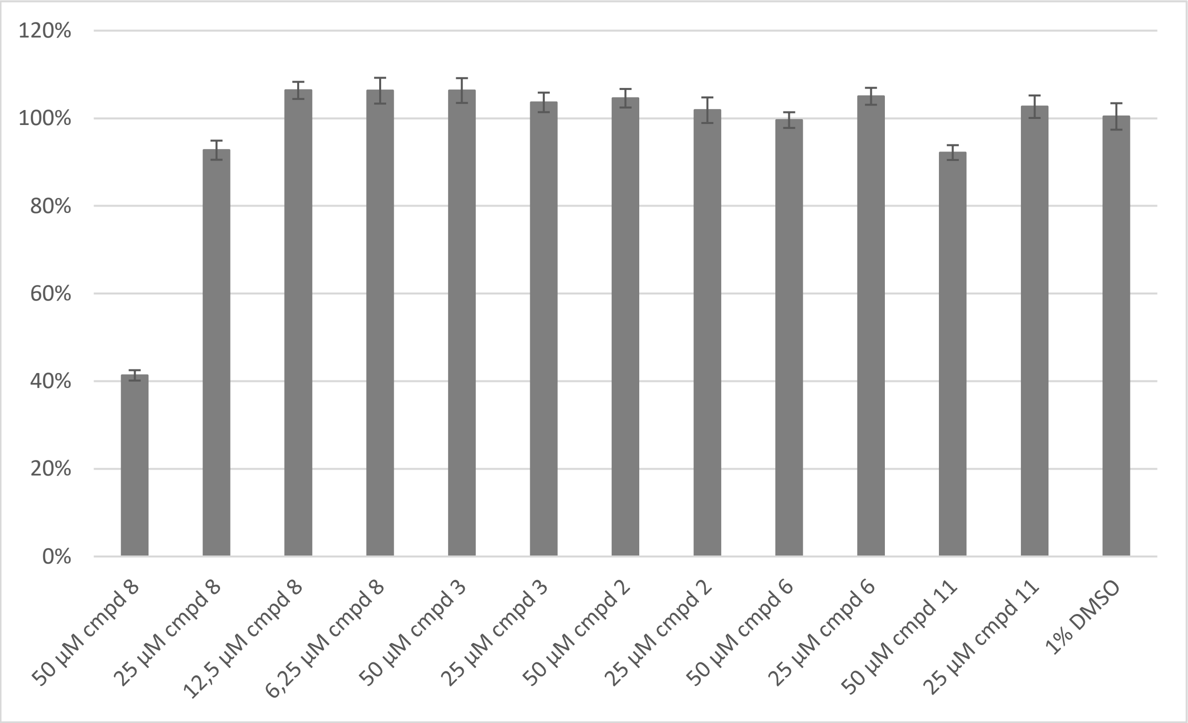

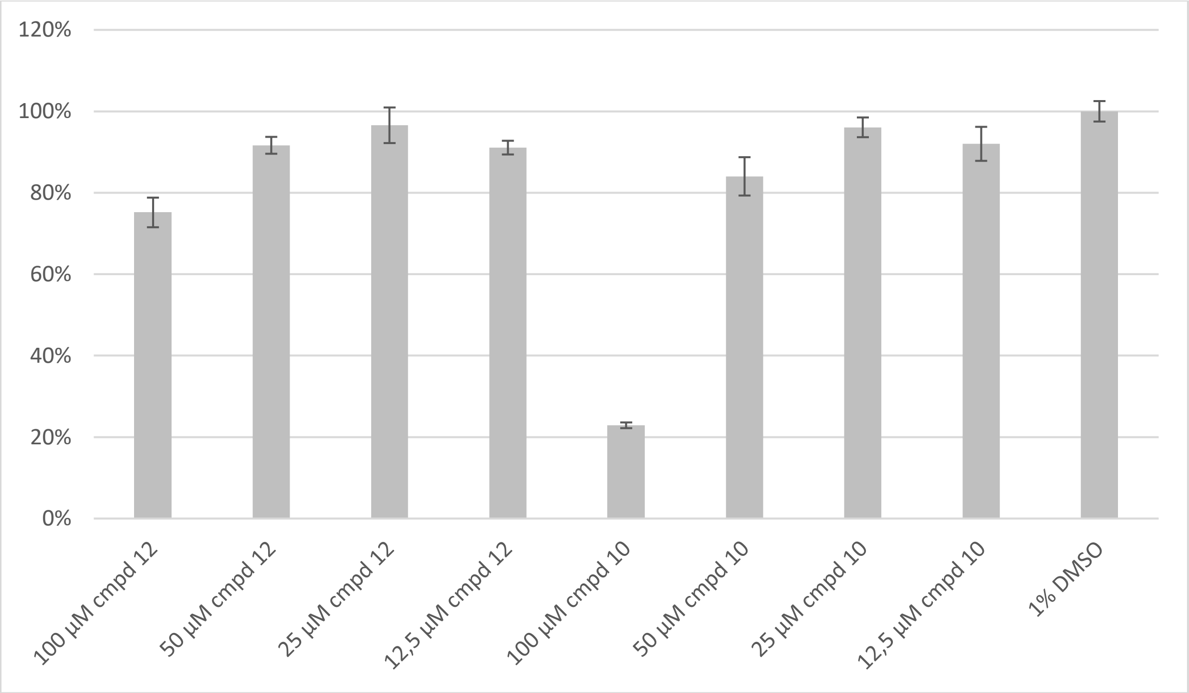

Figure S2. Toxity towards J774 cells after 6 h.

		ctrl	cmpd 8	cmpd 8	cmpd 8	cmpd x	cmpd x	cmpd x	cmpd y	cmpd y	cmpd y		
Plate 1	1	2	3	4	5	6	7	8	9	10	11	12	µM
A	45897	46712	1509	1580	1590	2402	2186	2448	2890	2122	2559	52157	100,0
B	50332	40243	1521	1733	1651	3980	4154	4652	23223	6083	6200	50714	50,0
C	51866	40643	1613	2008	1842	26774	31782	31862	37832	34600	37497	50704	25,0
D	38027	41897	14134	14853	21438	35243	32780	37926	39539	41327	45301	50896	12,5
E	1340	1693	28901	31184	31347	30403	34183	38190	36907	40086	42737	48173	6,3
F	1370	1588	37007	38574	43412	42090	40836	42744	43279	45837	45141	49104	3,1
G	1659	1562	42082	42533	41370	42613	42574	44876	47904	43074	44807	49384	1,6
H	1652	1704	36700	41964	41229	38978	34984	34179	35121	31927	34387	47764	-

Figure S3. Raw data of GFP measurement directly from plate reader showing the control strains PAK(pCS500) well B2-D2 and PAK*exsA*(pCS500) well E2-G2 and PAK(pCS500) with addition of compound 8 at 100-3.1 µM in triplicates B3-G5.

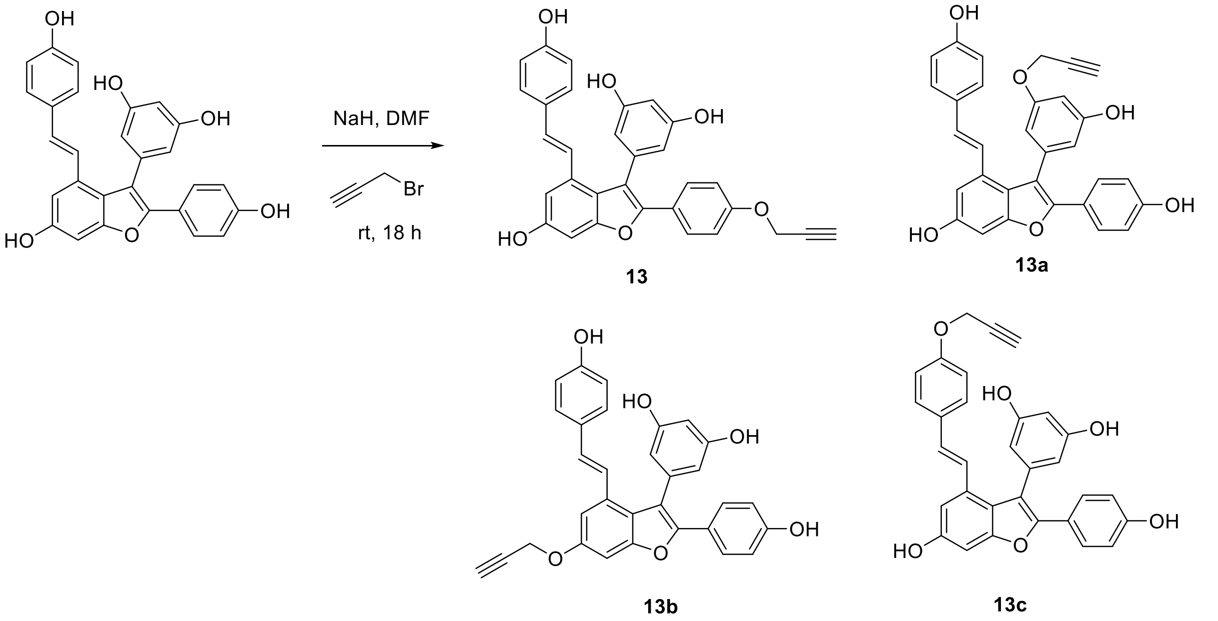

Figure S4. Synthesis of monopropargylated viniferifuran.

To a mixture of viniferifuran (120 mg, 0.26 mmol, 1 equiv.), TBAI (98 mg, 1 equiv.) in DMF (5 mL) at 0 °C, was added 60% NaH (32 mg, 3 equiv.). The reaction was stirred at 0 °C for 30 min and then a 80% weight solution of propargyl bromide in toluene (43 µL, 1.5 equiv.) was added to the reaction mixture. The mixture was stirred at rt for overnight and then neutralized with 1 N HCl. The mixture was extracted with ethyl acetate. The organic phase was washed with H_2O and brine, dried over $MgSO_4$, filtered and concentrated to give a crude. The crude was purified by high performance liquid chromatography using a reversed-phase C-18 column and a gradient of 20-100% MeOH in H_2O (both + 0.005% HCOOH) over 40 min to afford 4 major fractions of monoalkylated products whose structures were analysed by 2D NMR: 13c (fraction 1, 5 mg); 13 (fraction 2, 7 mg); mixture of 13a and 13 (fraction 3, 1 mg) and 13b (fraction 4, 5 mg). LCMS of mono propargylated product: ESI- [M-H]- for $C_{31}H_{21}O_6$ calculated 489.1, found 489.2.

Compound 13: ^{1}H NMR (600 MHz, Acetone-*d*$_6$) δ 8.52 (br s, 4H), 7.59 (d, *J* = 9.0 Hz, 2H), 7.13 (d, *J* = 2.0 Hz, 1H), 7.07 (d, *J* = 8.5 Hz, 2H), 7.02 (d, *J* = 16.3 Hz, 1H), 6.98 (d, *J* = 9.0 Hz, 2H), 6.96 (d, *J* = 16.3 Hz, 1H), 6.92 (d, *J* = 2.0 Hz, 1H), 6.74 (d, *J* = 8.6 Hz, 2H), 6.61 (t, *J* = 2.2 Hz, 1H), 6.50 (d, *J* = 2.2 Hz, 2H), 4.81 (d, *J* = 2.4 Hz, 2H), 3.08 (t, *J* = 2.4 Hz, 1H). ^{13}C NMR (150 MHz, Acetone-*d*$_6$) δ 161.5, 159.3, 159.1, 157.7, 157.0, 150.5, 138.9, 134.1, 131.1, 130.2, 129.7, 129.0, 126.2, 123.7, 123.0, 118.9, 117.2, 116.7, 110.8, 108.5, 104.2, 98.3, 80.6, 78.1, 57.3.

Compound 13b: ^{1}H NMR (600 MHz, Acetone-*d*$_6$) δ 8.52 (br s, 4H), 7.51 (d, *J* = 8.9 Hz, 2H), 7.25 (d, *J* = 2.1 Hz, 1H), 7.14 (d, *J* = 2.1 Hz, 1H), 7.09 (d, *J* = 8.5 Hz, 2H), 7.07 (d, *J* = 16.3 Hz, 1H), 7.03 (d, *J* = 16.3 Hz, 1H), 6.81 (d, *J* = 8.9 Hz, 2H), 6.75 (d, *J* = 8.5 Hz, 2H), 6.60 (t, *J* = 2.2 Hz, 1H), 6.50 (d, *J* = 2.2 Hz, 2H), 4.91 (d, *J* = 2.4 Hz, 2H), 3.12 (t, *J* = 2.4 Hz, 1H). ^{13}C NMR (150 MHz, Acetone-*d*$_6$) δ 161.5, 159.4, 159.1, 158.0, 156.5, 151.9, 138.8, 134.0, 131.1, 130.7, 129.7, 129.4, 124.4, 124.2, 123.4, 118.0, 117.3, 117.2, 110.9, 108.8, 104.2, 97.8, 80.9, 78.1, 58.0.

Compound 13c: ^{1}H NMR (600 MHz, Acetone-*d*$_6$) δ 8.54 (br s, 4H), 7.49 (d, *J* = 8.8 Hz, 2H), 7.17 (d, *J* = 8.6 Hz, 2H), 7.13 (d, *J* = 2.0 Hz, 1H), 7.08 (d, *J* = 16.3 Hz, 1H), 6.99 (d, *J* = 16.3 Hz, 1H), 6.92 (d, *J* = 2.4 Hz, 2H), 6.90 (d, *J* = 2.0 Hz, 1H), 6.80 (d, *J* = 8.8 Hz, 2H), 6.60 (td, *J* = 2.2 Hz,

1H), 6.50 (d, *J* = 2.2 Hz, 2H), 4.79 (d, *J* = 2.4Hz, 2H), 3.08 (t, *J* = 2.4 Hz, 1H). ^{13}C NMR (150 MHz, Acetone-*d*$_6$) δ 161.4, 159.3, 159.2, 157.5, 156.9, 151.2, 139.0, 133.6, 133.0, 129.6, 129.4, 129.3, 124.9, 124.3, 118.0, 117.2, 116.8, 110.9, 108.6, 104.1, 98.6, 80.7, 78.0, 57.3.

Mixture of compound 13a and 13 (see ^{1}H NMR spectrum below)

Compound 13, ^{1}H

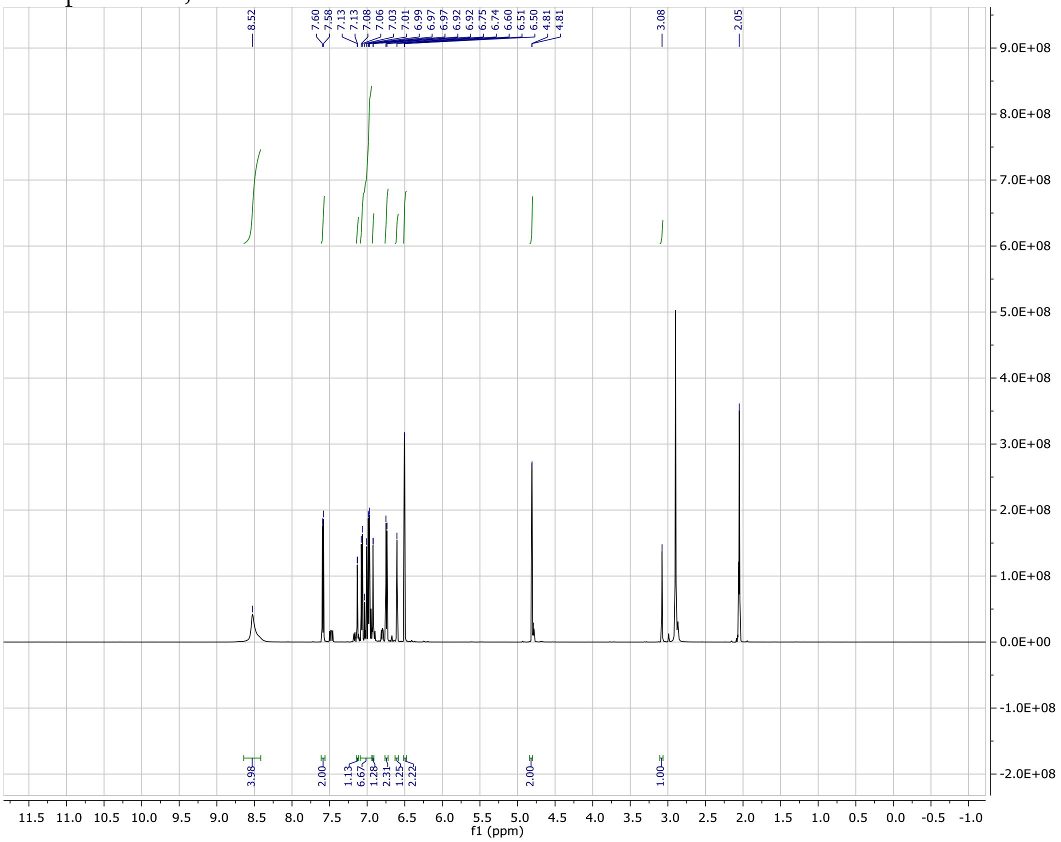

Compound 13, ^{13}C

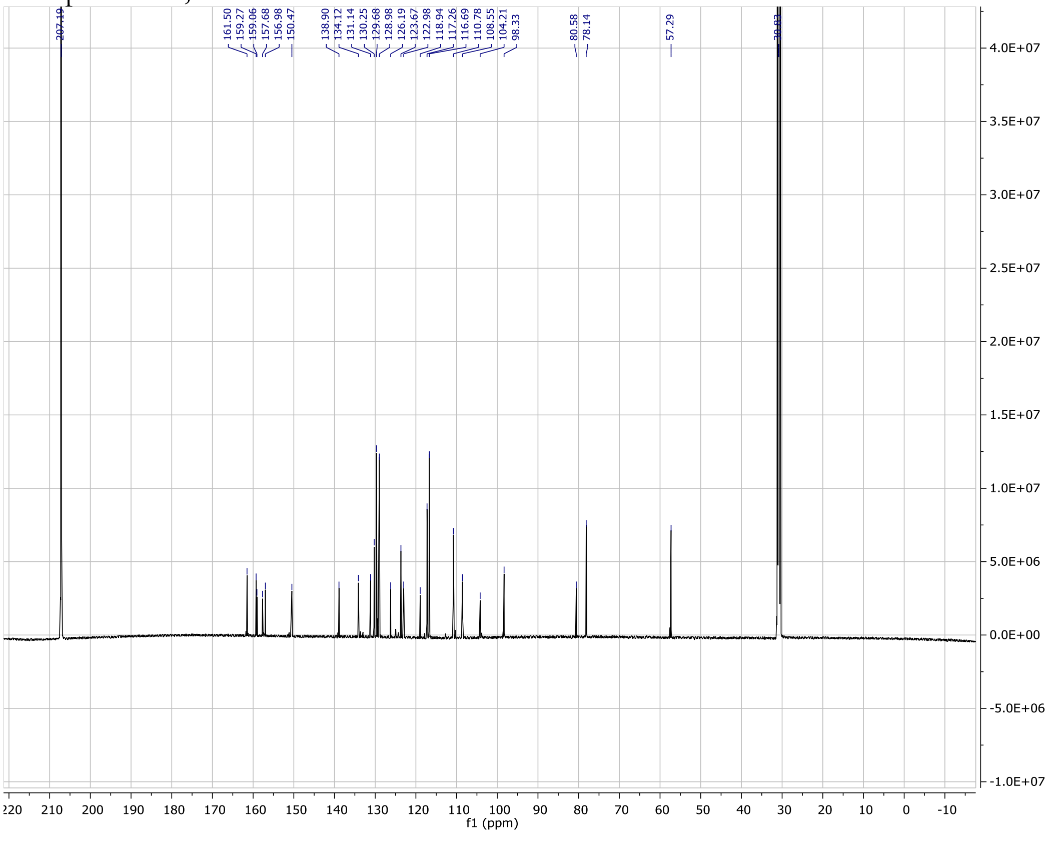

Compound 13b, ^{1}H

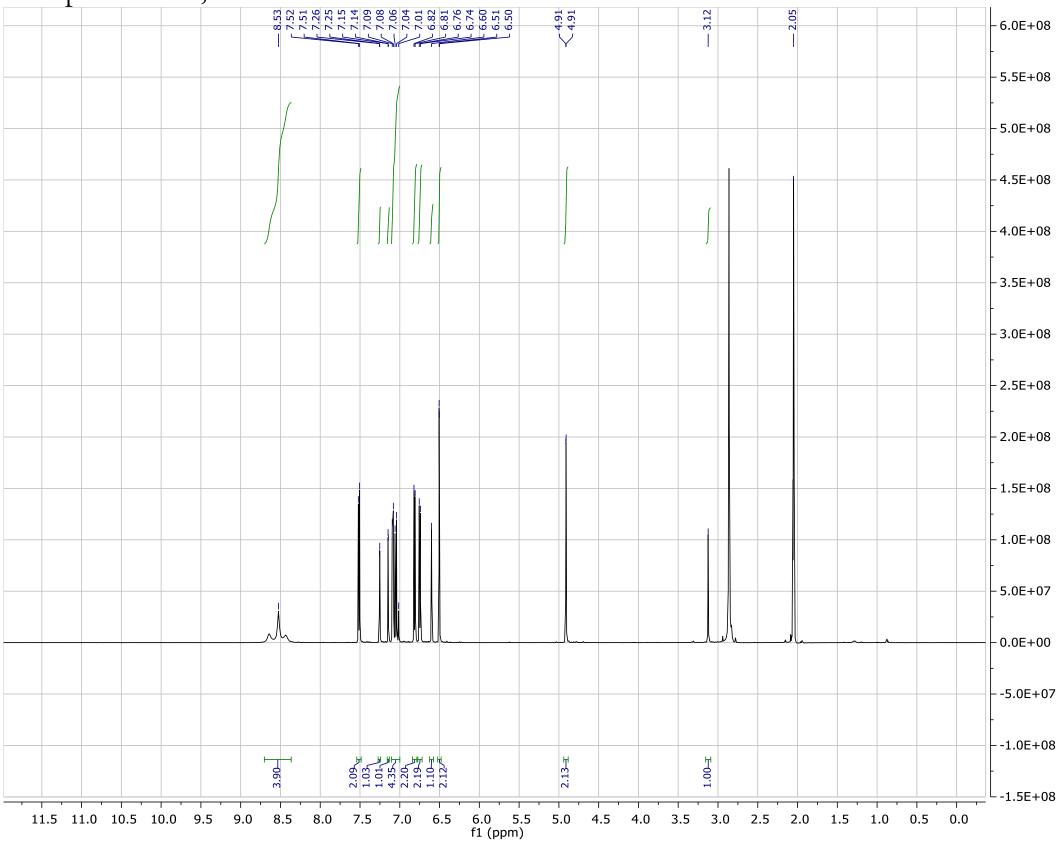

Compound 13b, ^{13}C

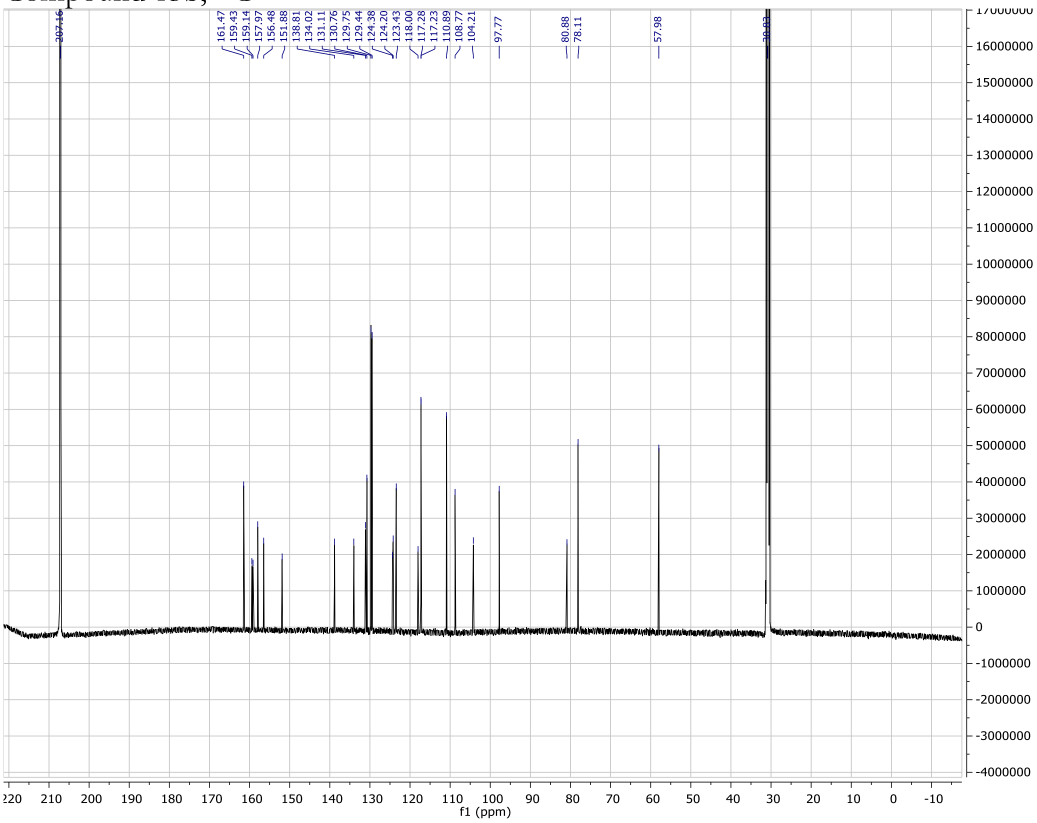

Compound 13c, ^{1}H

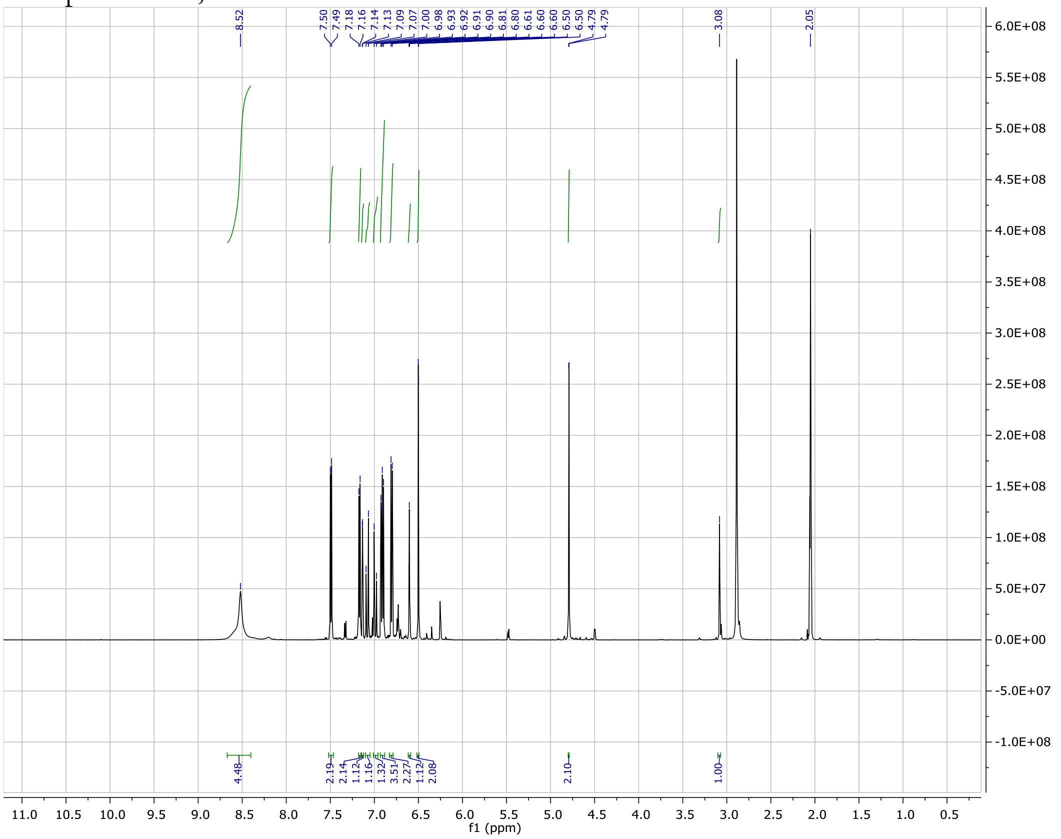

Compound 13c, ^{13}C

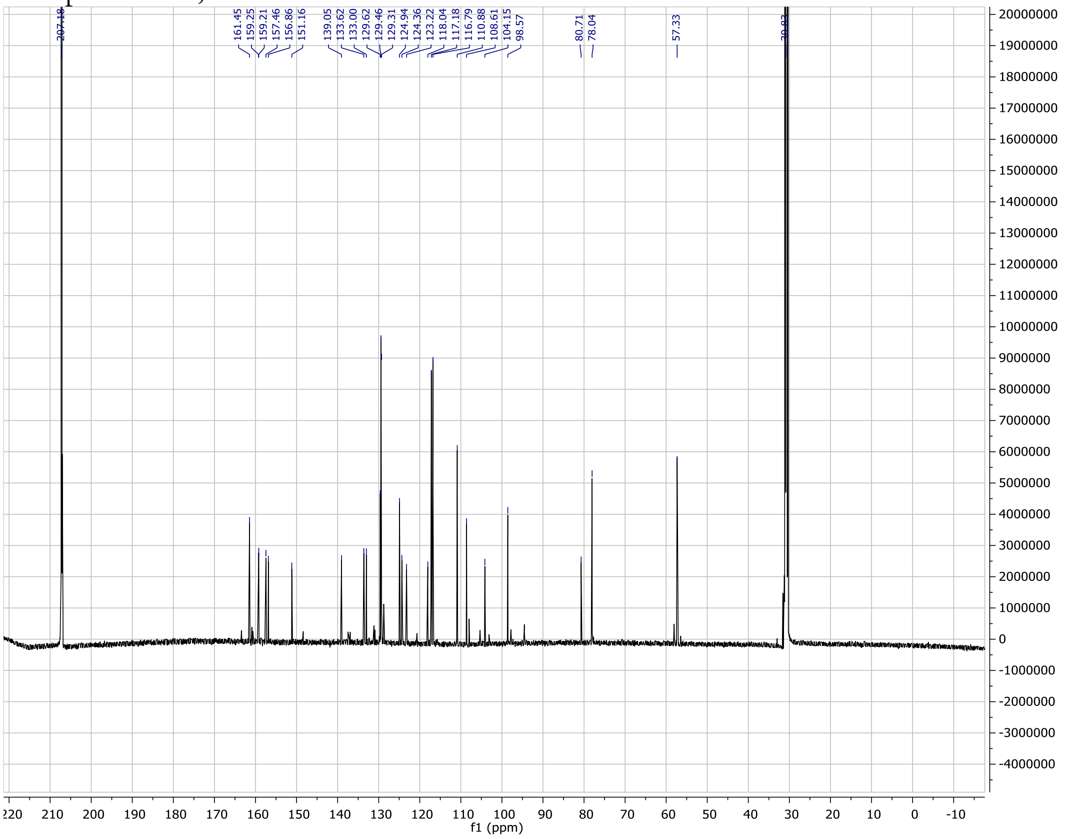

Mixture of compound 13a and 13, ^{1}H

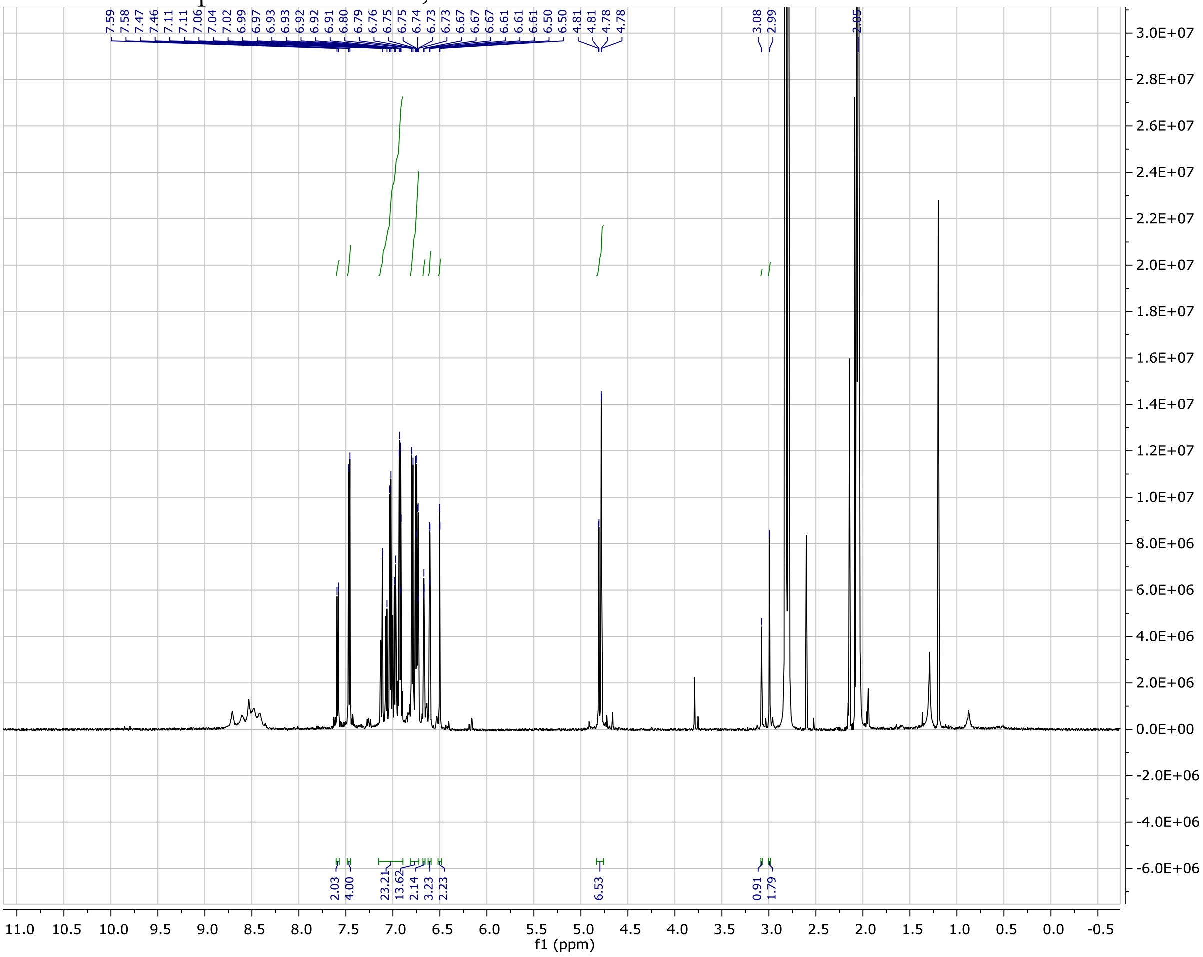

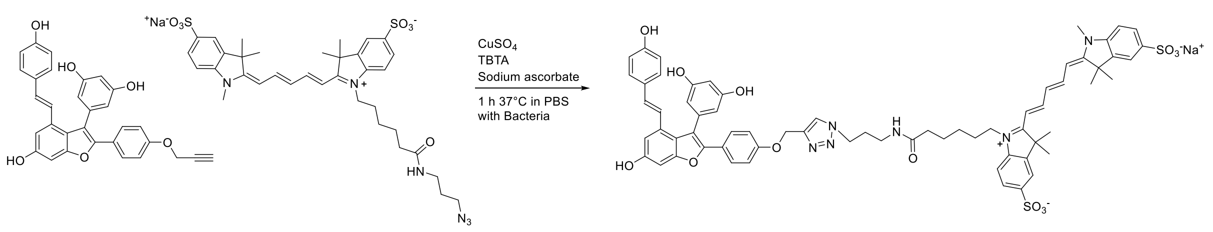

Figure S5. Click chemistry between compound 13 bound to bacteria and sulfonyl-cyanine azide.
